# Construction and Verification of a Risk Prediction Model for Suicidal Ideation in Patients With Bipolar Disorder: A Machine Learning Analysis

**DOI:** 10.31083/AP45589

**Published:** 2026-04-23

**Authors:** Xia Luo, Xiaoling Lin, Qinghua Zhao, Shaoyu Mu, Xueying Yu, Chenyun Zhang, Duoduo Lin, Tian Zhou, Qijun Shui

**Affiliations:** ^1^School of Nursing, Chongqing Medical University, 400016 Chongqing, China; ^2^School of Nursing, Xiamen Medical College, 361023 Xiamen, Fujian, China; ^3^Center of Nursing Research, The First Affiliated Hospital of Chongqing Medical University, 400042 Chongqing, China; ^4^Department of Nursing, The Third Affiliated Hospital of Sun Yat-sen University, 510630 Guangzhou, Guangdong, China; ^5^Department of Psychiatry, Xiamen Xianyue Hospital, Xianyue Hospital Affiliated with Xiamen Medical College, Fujian Psychiatric Center, Fujian Clinical Research Center for Mental Disorders, 361012 Fuzhou, Fujian, China; ^6^Department of Nursing, Affiliated Brain Hospital, Guangzhou Medical University, 510145 Guangzhou, Guangdong, China

**Keywords:** bipolar disorder, suicidal ideation, machine learning, random forest

## Abstract

**Background:**

Bipolar disorder (BD) is closely associated with suicidal ideation (SI). The development of an effective prediction model for SI in BD patients could facilitate early risk identification in high-risk groups.

**Methods:**

This study employed a cross-sectional design. Patients with BD were enrolled from three tertiary hospitals between July 2021 and July 2024. All participants were randomly allocated to training (n = 204) or testing (n = 88) sets at a 7:3 ratio. A hybrid feature selection strategy integrating the data-driven Boruta algorithm with clinical expertise was used to identify potential predictors of SI. Nine machine learning algorithms were applied to the training set to construct SI prediction models. The optimal model was selected through comprehensive evaluation of the area under the receiver operating characteristic curve (AUC), F1 score, balanced accuracy, sensitivity, and other indicators. SHapley Additive exPlanations (SHAP) analysis was used to rank and interpret the importance of features in the best-performing model and to assess their contributions to SI.

**Results:**

A total of 292 patients with BD were analyzed, of whom 149 (51.03%) reported SI during the past week. Among the nine models, the random forest (RF) model demonstrated superior predictive performance, with an AUC of 0.915 (95% CI: 0.850–0.965), a balanced accuracy of 0.818, a sensitivity of 0.891, a specificity of 0.833, a precision of 0.826, a average precision of 0.922, an F1 score of 0.860, and a Matthews correlation coefficient of 0.704. The SHAP analysis revealed that quality of life was the most influential predictor, followed by the number of depressive episodes, history of suicide attempts, cognitive functioning, and emotional abuse in childhood trauma.

**Conclusions:**

RF-based models can effectively predict SI in BD patients and inform clinically targeted interventions.

## Main Points

1. This cross-sectional study developed and compared nine machine learning models to predict suicidal ideation in 292 patients with bipolar disorder.

2. The random forest model demonstrated excellent predictive performance (area under the curve (AUC): 0.915) for identifying high-suicide-risk bipolar patients.

3. Quality of life emerged as the most significant predictor, followed by the number of depressive episodes, a history of suicide attempts, cognitive functioning (both subjective complaints and objective performance), and emotional abuse in childhood trauma.

## 1. Introduction

Suicide, defined as death caused by intentional self-directed harm, remains a leading cause of mortality worldwide, accounting for more than 700,000 deaths annually [1]. Suicidal ideation (SI), a precursor to suicidal behavior, involves self-reported inclinations toward self-harm without preparatory actions [2]. Recognized as the nascent phase in the progression toward suicide, SI is deemed a prerequisite for suicidal behavior and serves as a key predictor of future suicidal attempts (SAs), necessitating early intervention [3]. The risk of suicide is extraordinarily high among patients diagnosed with bipolar disorder (BD). Indeed, among psychiatric disorders, BD is linked to the highest suicide rates [4, 5, 6]. This risk is estimated to be 30–60 times greater than that observed in the general population [4, 5, 6]. Studies indicate that approximately 30%–50% of adult patients with BD attempt suicide, and approximately 15%–20% ultimately die by suicide [4, 5, 6]. SI is prevalent in patients with BD and appears to be a risk factor for completed suicide. Approximately 59% of patients with BD report SI, a proportion that is 20–30 times greater than that in the general population [7]. However, some studies conflate SI with SAs, limiting its clinical utility [8]. This conflation obscures distinct intervention pathways. Therefore, precisely identifying and addressing the risk factors for SI specifically among patients with BD are critical public health priorities for targeted prevention and intervention before suicidal behaviors escalate.

Prior studies have identified multiple risk factors for SI in patients with BD, such as a history of SAs, younger age, younger age at onset of illness, more severe depressive or manic symptoms, BD type II/nos, higher levels of insight and impulsiveness, concurrent hopelessness, a history of childhood trauma, neurocognitive dysfunction, functional impairment, a poor or dysfunctional family environment, and low psychological resilience, social support and quality of life (QoL) [9, 10, 11, 12, 13, 14, 15]. Crucially, for patients with BD, a previous SAs can powerfully predict future SI [13, 15], with 40% of suicide deaths occurring subsequent to such an attempt [15]. The risk of SI is particularly pronounced among younger patients with BD, especially within the first few years following the onset of the illness [15]. Aaltonen *et al*. [12] reported that younger age, severe depressive symptoms, BD type II/nos, hopelessness, and childhood physical abuse can independently predict SI in individuals with BD. Moreover, other studies have demonstrated that compared with manic and hypomanic phases, mixed and depressive episodes of BD are associated with a higher likelihood of SI [16]. Our previous research revealed that depressive symptoms and cognitive functioning are predictors of SI in patients with BD [14]. Furthermore, adverse family dynamics, such as excessively expressed emotions, reduced cohesion, and increased conflict, are correlated with an elevated risk of SI and SAs [10]. Notably, SI can mediate the relationship between family and psychosocial functioning [9]. Given the complex interplay among these risk factors for SI in patients with BD, the development of clinically actionable prediction tools is critical for enabling early interventions to reduce preventable suicide deaths.

Although existing suicide prediction models have achieved acceptable classification accuracy, their predictive utility remains constrained by methodological challenges. Traditional approaches often rely on univariate screening followed by logistic regression, which risk oversimplifying the complex, interactive nature of psychopathological data. These methods may introduce multicollinearity or overfitting while failing to capture the nonlinear dynamics inherent in high-dimensional clinical datasets [17]. Furthermore, they typically lack the ability to quantify the individual contribution of each risk factor to the outcome. Consequently, advanced analytical techniques that can more effectively integrate these multifaceted predictors are needed to develop robust clinical prediction tools. Machine learning (ML) is expected to address these constraints by algorithmically detecting multidimensional patterns within high-dimensional data [18]. By circumventing linearity assumptions and automatic feature weighting, ML techniques identify clinically significant variables while enhancing predictive accuracy, offering a methodological advance for SI risk modeling [18].

ML has emerged as a highly promising tool in psychiatric research because of its robust ability to integrate multidimensional predictors and classify outcomes with heightened precision [19]. Ensemble methods and deep learning architectures model complex nonlinear interactions and adapt to subtle data variability within high-dimensional clinical-behavioral data by capturing patterns eluding conventional approaches [20]. In addition, automated feature selection and scalable pattern recognition can efficiently address the complex risk factors associated with suicidality [17]. Furthermore, ML natively handles datasets comprising mixed feature types (continuous and categorical), which aligns with our predictor set comprising scales, counts, and binary indicators. The ensemble structure of methods such as Random Forest (RF), built on bagging and random feature selection, further confers robustness to noise and missing values. These techniques not only increase the precision of prediction models but also generate clinically actionable insights to guide targeted interventions and personalized health care.

ML models have been applied to predict SI or SAs in diverse populations. For example, Deng *et al*. (2025) [21] constructed risk prediction models for SAs in patients with mood disorders using the RF method, which exhibited good discriminant ability, stability, and calibration. In addition, ML algorithms have been utilized to predict SI among university students in Bangladesh (with more than 90% accuracy) [22] and among youth with autism spectrum disorder [23], demonstrating the potential of data-driven approaches for informing precision prevention strategies and guiding tailored support for high-risk subgroups.

To the best of our knowledge, research using ML models to predict SI among patients with BD is nearly nonexistent. Thus, this study aimed to develop ML-based prediction models for SI through multidimensional assessments of psychiatric, psychological, and sociodemographic variables in patients with BD.

## 2. Materials and Methods

### 2.1 Participants

This study used a cross-sectional design. The reporting of this study follows the Strengthening the Reporting of Observational Studies in Epidemiology (STROBE) guidelines for cross-sectional studies, for STROBE checklist see **Supplementary Material**. Patients with BD (aged 18–60 years) were recruited through convenience sampling from the psychiatric departments of three tertiary hospitals between July 2021 and July 2024. The sample size was determined based on the events per variable (EPV) criterion, with a minimum of 10 events per predictor variable, requiring at least 140 participants with SI. The diagnosis of BD was rigorously determined by two psychiatrists using the Structured Clinical Interview for DSM-V Axis I Disorders, Clinical version, along with the 17-item Hamilton Depression Rating Scale (HDRS-17) [24] and the Young Mania Rating Scale (YMRS) [25]. All patients received pharmacological treatment, with valproate, lithium, and antipsychotics representing the most commonly prescribed medications. All the participants had the ability to understand and read Chinese, and cooperate in completing the relevant tests.

Patients were excluded for any of the following: pregnancy/lactation; current severe depressive/manic episodes (HDRS-17 ≥24 or YMRS ≥20) or active psychotic symptoms that impeded testing; severe physical/neurological disorders (e.g., epilepsy, brain injury); history of head trauma with loss of consciousness >5 minutes; color blindness/uncorrected visual impairment; electroconvulsive therapy within six months; substance/alcohol abuse or dependence within one year (as per DSM-V criteria); intellectual disability (Wechsler Adult Intelligence Scale score <70); or participation in other interventional trials within three months prior to enrollment.

### 2.2 Assessments

In this study, multiple patient characteristics, such as sociodemographic and clinical indicators, SI within the past week, cognitive functioning (objective cognitive performance and subjective cognitive complaints), psychosocial factors (resilience, self-esteem, and childhood trauma), environmental influences (social support and family functioning), and functional outcomes (QoL), were assessed. The detailed measurement protocols are described below.

#### 2.2.1 Sociodemographic and Clinical Assessment

The sociodemographic and clinical data of all participants were collected using a structured questionnaire and medical records, such as demographic profile (age, sex, body mass index (BMI), waist‒hip ratio (WHR), smoking status, marital status, and occupation), disease characteristics (duration of untreated illness, course, number of depressive episodes, history of psychotic symptoms, history of SAs, depressive symptoms, and manic symptoms), treatment parameters (current pharmacotherapy regimens). Depressive and manic symptoms were assessed using the HDRS-17 and the YMRS, respectively.

Blood biomarker profiling was conducted for all participants’ standardized protocols. Fasting venous blood samples (5 mL) were collected at 07:00 by trained phlebotomists. The samples were immediately aliquoted into appropriate vacutainers and processed according to established laboratory protocols, such as centrifugation (3000 rpm, 10 min, 4 °C) and serum separation. The biochemical panel included hematological indices (red blood cell (RBC) count, white blood cell (WBC) count, and hemoglobin (HGB), hepatic function (glutamic oxaloacetic transaminase (AST), glutamic-pyruvic transaminase (ALT), albumin (ALB), total protein (TP), globulin (GLO), and total bilirubin (TBIL)), and metabolic markers (blood glucose (BG), cholesterol (CHO), triglyceride (TG), high-density lipoprotein cholesterol (HDL), low-density lipoprotein cholesterol (LDL), apolipoprotein A1 (APOA1), and apolipoprotein B (APOB)). All analyses were performed in Clinical Laboratory Improvement Amendments (CLIA)-certified laboratories using automated analyzers with strict quality-control measures.

#### 2.2.2 Suicidal Ideation

SI was evaluated using the first five items of the Beck Scale for Suicidal Sdeation (BSI) [26]. Each item is scored on a 3-point Likert scale ranging from 0 to 2. Participants with a cumulative score of ≥1 across these five items were classified as having experienced SI during the preceding week, while a score of 0 indicated no SI. The validated BSI demonstrated acceptable internal consistency in the Chinese population (Cronbach’s α = 0.68) [27].

#### 2.2.3 Cognitive Functioning

Objective cognitive functioning was evaluated using a battery of neuropsychological tests. Attention and processing speed were assessed using the Trail Making Test Part A [28], Digital Span Forward subtest, and Digit Symbol Coding subtest of the Wechsler Adult Intelligence Scale-Revised by China (WAISRC) [29]. Memory was evaluated using the Digital Span Backward subtest of the WAISRC [29] and the Visual Reproduction and Recognition subtest of the Wechsler Memory Scale-Revised [30]. Executive functions were assessed using the Trail Making Test Part B [31], a categorical verbal fluency test (animal naming) [32], and the Stroop Color and Word Test [33]. The raw scores were converted into standardized z scores. The mean of the z scores from the respective subtests was computed to generate the composite domain scores. The global cognitive functioning score was calculated by averaging all the domain scores [34].

Subjective cognitive complaints were detected using the Cognitive Complaints in Bipolar Disorder Rating Assessment (COBRA) [35]. The COBRA is a 16-item unidimensional instrument answered on a 4-point Likert scale ranging from 0 to 3. A total score ≥11 points indicated clinically significant cognitive complaints. The Chinese version of the COBRA has good validity and reliability (Cronbach’s α = 0.91) [36].

#### 2.2.4 Psychosocial Variables

Psychological resilience over the preceding two-week period was evaluated using the Resilience Questionnaire for Bipolar Disorder (RBD). The RBD comprises 23 items evaluated on a 5-point Likert scale ranging from 1 (strongly disagree) to 5 (strongly agree). Higher scores indicate greater resilience. The Chinese version of the RBD has good reliability and validity, with a Cronbach’s α value of 0.95 [37].

The self-esteem level of the subjects was assessed using the Rosenberg Self-Esteem Scale (RSES). The scale contains 10 items and employs a 4-point Likert scale ranging from 1 (strongly disagree) to 4 (strongly agree). Higher scores reflect a greater level of self-esteem. The Chinese version of the RSES has good validity (Cronbach’s α = 0.92) [38].

The traumatic experiences of subjects up to 16 years of age were evaluated using the Childhood Trauma Questionnaire (CTQ) [39]. The CTQ includes five dimensions: emotional neglect, emotional abuse, physical neglect, physical abuse, and sexual abuse. The CTQ has 28 items and uses a 5-point scale ranging from 1 (never) to 5 (always), with higher scores indicating more severe traumatic experiences. The Chinese version of the CTQ has good reliability and validity (Cronbach’s α = 0.77) [40].

#### 2.2.5 Environmental Variables

Social support in the past year was assessed using the Social Support Rating Scale (SSRS). The SSRS consists of ten items, in three dimensions: subjective support, objective support, and utilization of support. The total scores range from 12 to 66, with higher scores indicating higher levels of social support. The item consistency of the SSRS ranged from 0.89 to 0.94, indicating good test-retest reliability [41].

Family functioning was evaluated through the general functioning dimension of the Family Assessment Device (FAD). A 4-point Likert scale was used, ranging from 1 (much like my home) to 4 (not at all like my home). Higher scores indicate healthier families. This scale has high reliability and validity, with the Cronbach’s α of each subscale ranging from 0.72 to 0.92 [42].

#### 2.2.6 Functional Outcomes

QoL was assessed using the 12-Item Short Form Health Survey (SF-12). The total score ranges from 12 to 48, with higher scores indicating better QoL. The SF-12 has good reliability and validity in the Chinese population (Cronbach’s α = 0.91) [43].

### 2.3 Statistical Analysis 

Statistical analyses and data visualization were conducted in Python 3.11.4 using standard scientific libraries (NumPy, SciPy, Matplotlib, etc.). The code development and execution were performed using Visual Studio Code 1.85. Missing data were handled by multiple imputations using the MICE package (version 1.0.2, https://pypi.org/project/mice/). Prior to model training, all the continuous variables were standardized using Z-score normalization (mean = 0, standard deviation = 1) to mitigate the influence of varying scales and units. The normality assumption for continuous variables was evaluated using the Shapiro–Wilk test, with descriptive statistics presented as the means (M) ± standard deviation (SDs) for normally distributed data and medians (Mdns) with first and third quartiles (Q1, Q3) for nonnormal distributions. Categorical variables are summarized using frequencies (n) and proportions (%). Group differences in sociodemographic, clinical, and psychological characteristics were analyzed using the independent samples *t* test for normally distributed continuous variables, the Mann‒Whitney U test for nonnormally distributed continuous variables, and Pearson’s χ^2^ test for categorical variables. Statistical significance was defined as a two-tailed *p*-value of < 0.05.

RF is a supervised ML algorithm based on ensemble learning that addresses classification and regression problems through bagging integration and optimization of decision trees, demonstrating efficacy in psychiatric prediction tasks. In addition to RF, eight other ML algorithms were utilized: logistic regression (LR), extreme gradient boosting (XGBoost), light gradient boosting machine (LightGBM), categorical boosting (CatBoost), gradient boosting machine (GBM), adaptive boosting (AdaBoost), extremely randomized trees (ET), and support vector classification (SVC).

The dataset was randomly partitioned into training (n = 204) and test (n = 88) sets at a ratio of 7:3 using the train_test_split tool in the sklearn module (version 1.7.1, https://pypi.org/project/scikit-learn/). A hybrid feature selection strategy combining data-driven technology (Boruta) and clinical expertise was employed to screen features that might be related to SI as predictors. This approach ensured the retention of both statistically significant and clinically relevant variables. All feature selection procedures were conducted solely on the training set to prevent information leakage from the test set.

In this study, all the ML models were constructed using their default hyperparameters to ensure a straightforward and equitable baseline comparison of algorithmic performance. Model development employed a stratified 5-fold cross-validation framework to ensure robust performance estimation. The model evaluation metrics included the area under the receiver operating characteristic curve (AUC-ROC), balanced accuracy (overall correctness), sensitivity (recall), specificity, precision, average precision, F1 score (harmonic mean of precision and recall), and the Matthews correlation coefficient (MCC, accounting for class imbalance). Class weighting was applied to address potential outcome class imbalances. The SHapley Additive exPlanations (SHAP) package was used to conduct an interpretive analysis of the model with the best prediction performance. The average |SHAP| value of the characteristic parameters was defined as their predictive importance for the ranking.

The comprehensive performance of the optimal model was evaluated using the learning curve, validation curve, and precision‒recall (PR) curve. First, the learning curve was used to analyze the evolution of both training and cross-validation scores as the size of the training dataset progressively increased to assess the learning behavior of the model and potential overfitting. The validation curve was subsequently used to evaluate the sensitivity of the model to the key hyperparameter (n_estimators) within the RF architecture. By mapping the performance metrics across incrementally adjusted estimator counts, this curve pinpoints the threshold value beyond which incremental performance gains plateaued. The PR curve is used to plot the precision against the recall to specifically evaluate the performance of the model across different classification thresholds. The area under the PR curve, quantified as the average precision score, provided a consolidated metric summarizing the precision‒recall trade-off quality across all the thresholds. Finally, for practical deployment and user interaction, an interactive web application was deployed. By leveraging the ExplainerDashboard framework (version 0.5.8, https://pypi.org/project/explainerdashboard/) (xpl.run_app()), a browser-accessible interface was delivered to enable the prediction of the SI based on inputs for relevant risk factors for patients.

The development of prediction models strictly followed the Transparent Reporting of a Multivariable Prediction Model for Individual Prognosis or Diagnosis (TRIPOD) statement guidelines. This framework integrates contemporary ML techniques with rigorous statistical practice. Clinical relevance was preserved through domain expert-guided feature selection, ensuring both the methodological robustness and translational applicability of the findings.

## 3. Results

### 3.1 Participant Characteristics

**Fig. 1.**
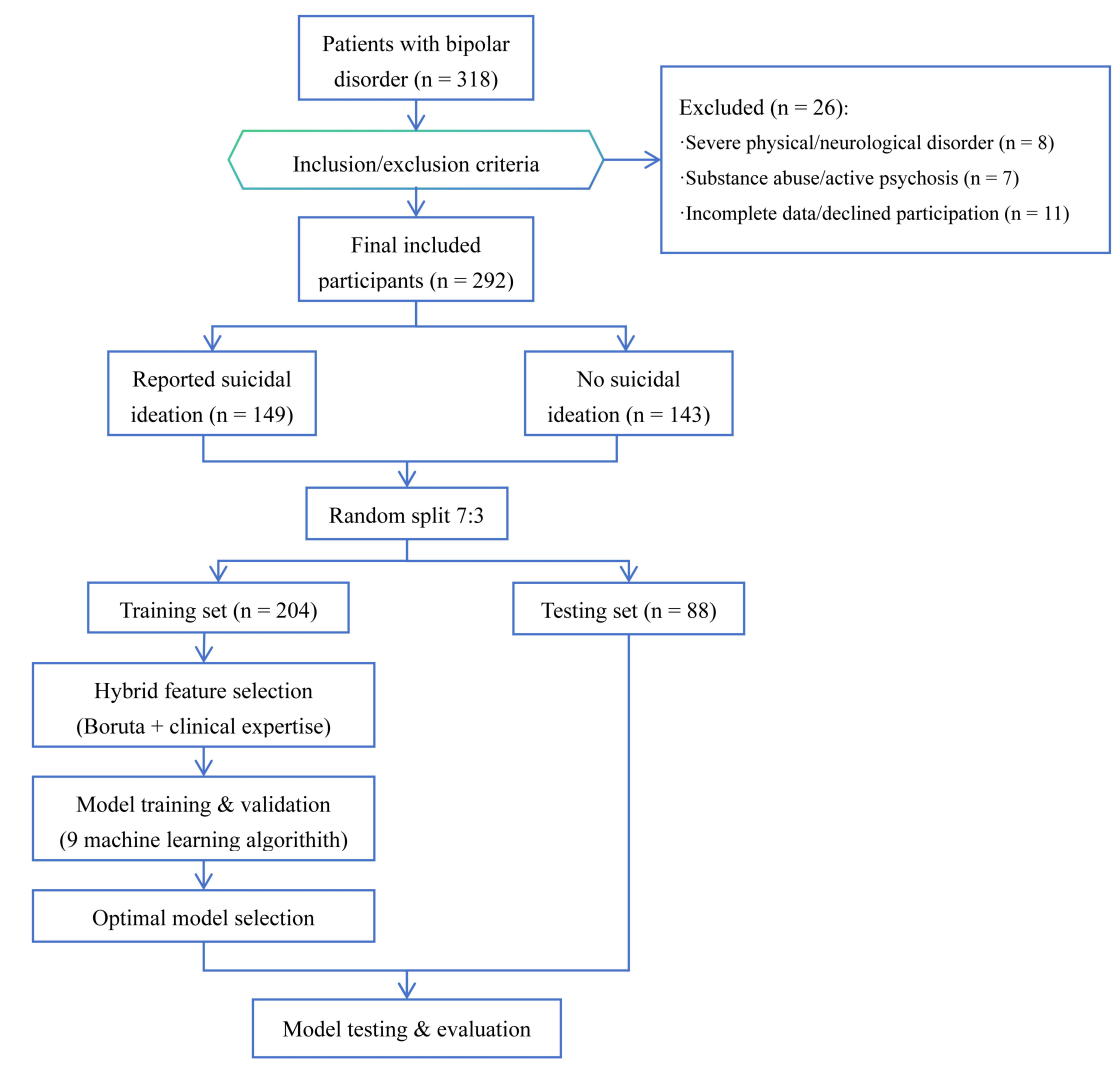
**Flowchart of participant selection and model development**.

Fig. 1 shows the participant flow diagram. During the study period, 318 patients with BD were assessed for eligibility. After applying inclusion/exclusion criteria, 292 patients were included in the final analysis. Among them, 149 (51.03%) reported SI within the past week, and 125 (42.81%) had a history of SAs. The mean patient age was 29.50 ± 11.90 years. Compared with the NSI group, the SI group was significantly younger (Cohen’s *d* = 0.39, *p* = 0.001), had a greater proportion of nonsmokers (Cramer’s V = 0.13, *p* = 0.007), and had a markedly greater prevalence of previous SAs (Cramer’s V = 0.33, *p* < 0.001). Comprehensive demographic characteristics, clinical profiles, and psychological assessments are presented in Tables 1,2, respectively.

**Table 1.** **Sociodemographic, clinical characteristics and inter-group comparison of bipolar patients with and without suicidal ideation**.

Variables		Total (n = 292)	SI (n = 149)	NSI (n = 143)	*t/χ^2^/U*	*p*
Age		29.50 ± 11.90	27.28 ± 11.66	31.82 ± 11.74	3.318	0.001^**^
Sex, n (%)	Male	104 (35.62)	45 (30.20)	59 (41.26)	3.423	0.064
	Female	188 (64.38)	104 (69.80)	84 (58.74)		
BMI, n (%)	<18.5	135 (46.23)	39 (26.17)	28 (19.58)	2.980	0.225
	18.5~24.0	90 (30.82)	70 (46.98)	65 (45.45)		
	≥24.0	67 (22.95)	40 (26.85)	50 (34.97)		
WHR, n (%)	Low	173 (59.25)	32 (21.48)	24 (16.78)	3.006	0.223
	Normal	63 (21.58)	36 (24.16)	27 (18.88)		
	High	56 (19.18)	81 (54.36)	92 (64.34)		
Smoking, n (%)	No smoking	201 (68.84)	114 (76.51)	87 (60.84)	9.858	0.007^**^
	Used to smoke	47 (16.10)	21 (14.09)	26 (18.18)		
	Current smoking	44 (15.07)	14 (9.40)	30 (20.98)		
Marriage, n (%)	In marriage	86 (29.45)	36 (24.16)	50 (34.97)	3.596	0.058
	Not in marriage	206 (70.55)	113 (75.84)	93 (65.03)		
Occupation, n (%)	Employed	228 (78.89)	32 (21.48)	29 (20.28)	0.012	0.914
	Unemployed	61 (21.11)	117 (78.52)	114 (79.72)		
Course		7.58 ± 8.02	7.10 ± 7.89	8.07 ± 7.89	1.050	0.295
Duration of Untreated Time, Mdn (Q1, Q3)		0 (0, 2)	1 (0, 2)	0 (0, 1)	–2.779^a^	0.005^**^
No. of depressive episodes, Mdn (Q1, Q3)		2 (1, 5)	2 (0, 3)	0 (0, 1)	–3.701^a^	<0.001^*⁣**^
Depressive symptom, n (%)	None	158 (59.18)	77 (51.68)	106 (74.13)	20.635	<0.001^*⁣**^
	Mild	69 (25.84)	40 (26.85)	29 (20.28)		
	Moderate or severe	40 (14.98)	32 (21.48)	8 (5.59)		
Manic symptom, n (%)	None	155 (58.05)	82 (55.03)	98 (68.53)	5.634	0.060
	Mild	69 (25.84)	41 (27.52)	28 (19.58)		
	Moderate or severe	43 (16.11)	26 (17.45)	17 (11.89)		
History of psychotic symptoms, n (%)	Yes	133 (56.12)	49 (32.89)	55 (38.46)	0.761	0.383
	No	104 (43.88)	100 (67.11)	88 (61.54)		
History of suicide attempts, n (%)	Yes	125 (42.81)	88 (59.06)	37 (25.87)	31.483	<0.001^*⁣**^
	No	167 (57.19)	61 (40.94)	106 (74.13)		
RBC (10^12^/L)		4.90 ± 1.43	4.65 ± 0.91	5.17 ± 1.58	3.490	0.001^*⁣**^
WBC (10^9^/L)		7.29 ± 3.80	7.26 ± 3.20	7.32 ± 4.36	0.117	0.907
HGB (g/L)		128.67 ± 17.58	127.25 ± 15.65	130.15 ± 19.33	1.414	0.158
AST (U/L)		19.28 ± 8.64	18.86 ± 8.92	19.73 ± 8.33	0.861	0.390
ALT (U/L)		18.46 ± 14.45	17.23 ± 13.03	19.73 ± 15.74	1.480	0.140
BG (mmol/L)		4.77 ± 1.09	4.80 ± 1.03	4.74 ± 0.94	–0.466	0.642
Ca^2+^ (mmol/L)		2.49 ± 1.38	2.43 ± 0.16	2.55 ± 1.80	0.840	0.401
ALB (g/L)		42.33 ± 5.27	42.76 ± 3.62	41.88 ± 5.80	–1.576	0.116
TP (g/L)		68.33 ± 7.40	68.61 ± 4.89	68.05 ± 9.33	–0.651	0.516
GLO (g/L)		26.39 ± 4.34	25.97 ± 3.64	26.82 ± 4.21	1.852	0.065
TBIL (µmol/L)		9.05 ± 4.74	8.62 ± 4.18	9.51 ± 4.40	1.780	0.076
CHO (mmol/L)		4.61 ± 3.89	4.32 ± 0.80	4.92 ± 5.01	1.444	0.150
TG (mmol/L)		1.34 ± 0.82	1.28 ± 0.80	1.40 ± 0.70	1.334	0.183
HDL (mmol/L)		1.28 ± 0.62	1.29 ± 0.74	1.26 ± 0.31	–0.580	0.562
LDL (mmol/L)		2.63 ± 0.72	2.63 ± 0.62	2.63 ± 0.69	0.051	0.959
APOA1 (g/L)		1.29 ± 0.81	1.26 ± 0.21	1.32 ± 1.04	0.696	0.487
APOB (g/L)		0.77 ± 0.22	0.78 ± 0.17	0.77 ± 0.23	–0.592	0.554
SBP (mmHg)		116.53 ± 13.31	115.21 ± 9.87	117.90 ± 11.43	2.156	0.032^*^
DBP (mmHg)		75.06 ± 9.39	73.96 ± 6.86	76.21 ± 8.11	2.572	0.011^*^

Note: SI, have suicidal ideation for nearly a week; NSI, no suicidal ideation for nearly a week; RBC, red blood cell; WBC, white blood cell; HGB, hemoglobin; AST, aspartate aminotransferase; ALT, alanine aminotransferase; BG, blood glucose; ALB, albumin; TP, total protein; GLO, globulose; TBIL, total bilirubin; CHO, cholesterol; TG, triglyceride; HDL, high-density lipoprotein cholesterol; LDL, low-density lipoprotein cholesterol; APOA1, apolipoprotein A1; APOB, apolipoprotein B; SBP, systolic blood pressure; DBP, diastolic blood pressure; BMI, body mass index; WHR, waist-hip ratio. The descriptive statistics and inter-group comparisons are presented based on the original dataset before multiple imputation. ^a^: Wilcoxon rank-sum test. ^*^: *p* < 0.05, ^**^: *p* < 0.01, ^*⁣**^: *p* < 0.001. M ± SDs: means ± standard deviations; Mdn (Q1, Q3): medians (first and third quartiles); n (%): frequencies (proportions).

**Table 2.** **Multidimensional factors and inter-group comparisons of bipolar patients with and without suicidal ideation**.

Variables			Total (n = 292)	SI (n = 149)	NSI (n = 143)	*t*	*p*
Cognitive functioning							
	Global objective cognitive functioning		0.12 ± 0.65	0.24 ± 0.63	0.01 ± 0.65	1.741	0.016^*^
		Attention and processing speed	0.02 ± 0.87	0.12 ± 0.82	–0.10 ± 0.92	–2.160	0.032^*^
		Visual memory	0.01 ± 0.68	0.03 ± 0.69	–0.02 ± 0.66	–0.601	0.548
		Executive functions	–0.02 ± 0.69	0.04 ± 0.69	–0.07 ± 0.68	–1.451	0.148
	Subjective cognitive complaints (yes/no)		192/100	121/28	71/72	30.885	<0.001^*⁣**^
Psychosocial variables							
	Resilience		91.79 ± 16.62	88.53 ± 17.60	95.19 ± 14.86	3.484	0.001^**^
	Self-esteem		25.30 ± 3.51	25.13 ± 4.20	25.48 ± 2.60	0.852	0.395
	Childhood trauma						
		Emotional neglect	2.65 ± 1.21	2.89 ± 1.21	2.40 ± 1.15	–3.539	<0.001^*⁣**^
		Emotional abuse	1.96 ± 0.96	2.19 ± 1.05	1.71 ± 0.79	–4.463	<0.001^*⁣**^
		Physical neglect	2.03 ± 0.79	2.14 ± 0.78	1.91 ± 0.78	–2.517	0.012^*^
		Physical abuse	1.62 ± 0.90	1.74 ± 0.95	1.50 ± 0.82	–2.329	0.021^*^
		Sexual abuse	1.40 ± 0.72	1.41 ± 0.74	1.39 ± 0.71	–0.210	0.834
Environmental variables							
	Social support		35.90 ± 8.55	33.32 ± 8.27	38.58 ± 8.01	5.513	<0.001^*⁣**^
	Family functioning						
		Problem solving	2.22 ± 0.55	2.27 ± 0.57	2.16 ± 0.53	–1.795	0.074
		Communication	2.42 ± 0.42	2.51 ± 0.43	2.33 ± 0.39	–3.632	<0.001^*⁣**^
		Roles	2.36 ± 0.36	2.41 ± 0.39	2.31 ± 0.32	–2.397	0.017^*^
		Affective responsiveness	2.48 ± 0.44	2.56 ± 0.46	2.40 ± 0.41	–3.170	0.002^**^
		Affective involvement	2.50 ± 0.42	2.54 ± 0.44	2.45 ± 0.40	–1.948	0.052
		Behavior control	2.41 ± 0.33	2.45 ± 0.34	2.38 ± 0.31	–1.862	0.064
		General functioning	2.33 ± 0.43	2.42 ± 0.43	2.23 ± 0.40	–3.769	<0.001^*⁣**^
Functional outcomes							
	Quality of life		32.88 ± 7.51	29.83 ± 6.90	36.07 ± 6.78	7.797	<0.001^*⁣**^

Note: SI, have suicidal ideation for nearly a week; NSI, no suicidal ideation for nearly a week. ^*^: *p* < 0.05, ^**^: *p* < 0.01, ^*⁣**^: *p* < 0.001.

### 3.2 Model Performance

**Fig. 2.**
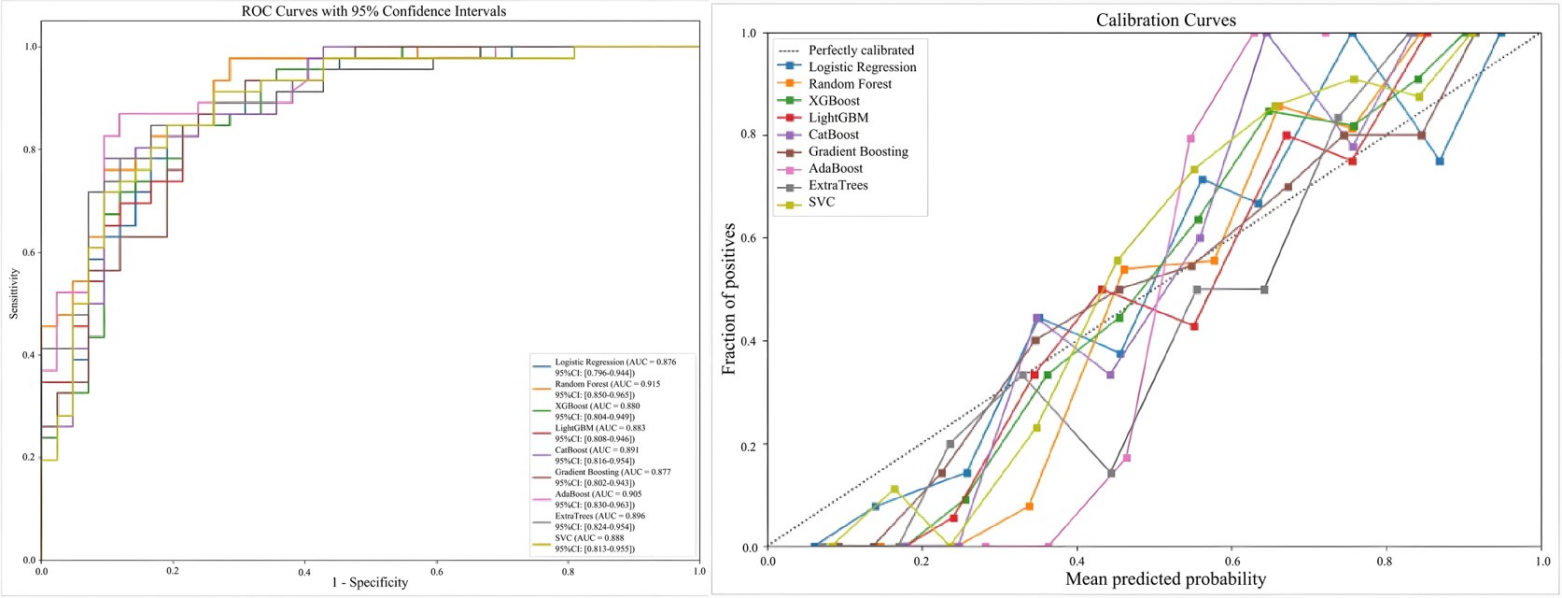
**ROC curves and calibration curves for nine machine learning models**. ROC, receiver operating characteristic.

The comparative performance metrics of the nine ML models are listed in Table 3, and the receiver operating characteristic (ROC) and calibration curves are presented in Fig. 2. All nine models achieved statistically significant AUC values (*p* < 0.001), among which the RF model outperformed the others across multiple validation metrics (Table 3), demonstrating superior discriminative capacity (AUC = 0.915; 95% CI: 0.850–0.965) and balanced accuracy (0.818). While AdaBoost approached RF performance in terms of AUC (0.905) and average precision (0.917) and ExtraTrees matched its sensitivity (0.891), RF maintained comprehensive superiority, establishing it as the optimal predictive architecture for the identification of SI.

**Table 3.** **Comparison of the performance of nine machine learning models**.

Model name	AUC (95% CI)	Balance	Sensitivity	Specificity	Precision	Average	F1	MCC
		Accuracy				Precision	Score	
Logistic Regression	0.876 (0.796–0.944)	0.807	0.804	0.810	0.822	0.879	0.813	0.613
Random Forest	0.915 (0.850–0.965)	0.818	0.891	0.833	0.826	0.922	0.860	0.704
XGBoost	0.880 (0.804–0.949)	0.806	0.826	0.786	0.809	0.876	0.817	0.613
LightGBM	0.883 (0.808–0.946)	0.773	0.761	0.786	0.795	0.890	0.778	0.546
CatBoost	0.891 (0.816–0.954)	0.818	0.826	0.810	0.826	0.887	0.826	0.636
Gradient Boosting	0.877 (0.802–0.943)	0.791	0.826	0.690	0.759	0.875	0.820	0.597
AdaBoost	0.905 (0.830–0.963)	0.851	0.870	0.810	0.851	0.917	0.826	0.636
ExtraTrees	0.896 (0.824–0.954)	0.803	0.891	0.714	0.774	0.909	0.828	0.618
SVC	0.888 (0.813–0.955)	0.829	0.848	0.810	0.830	0.886	0.839	0.658

Note: XGBoost, extreme Gradient Boosting; LightGBM, Light Gradient Boosting Machine; CatBoost, Categorical Boosting; AdaBoost, Adaptive Boosting; ExtraTrees, Extremely Randomized Trees; SVC, Support Vector Classifier; AUC, area under the curve; MCC, Matthews Correlation Coefficient.

### 3.3 Variable Importance

**Fig. 3.**
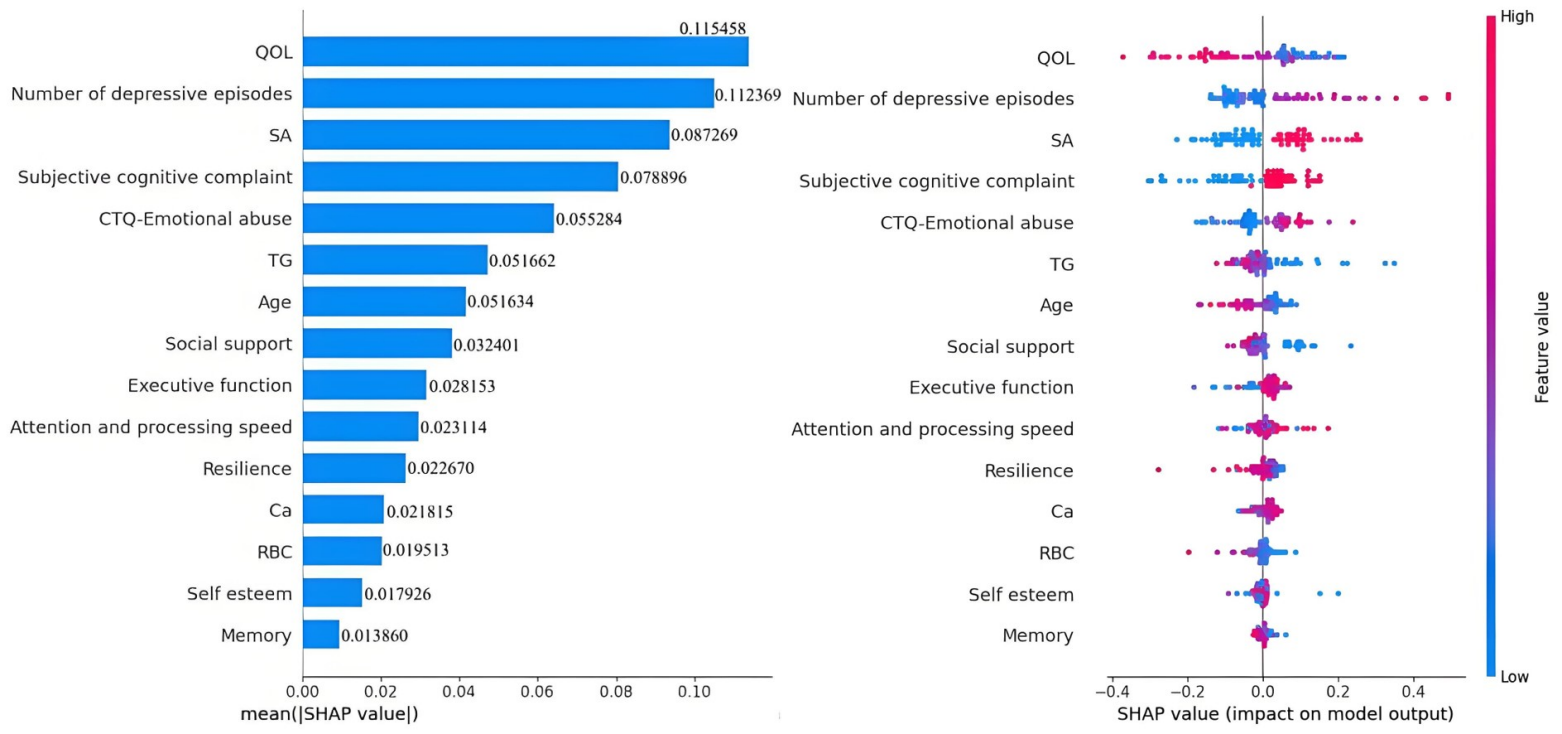
**The variable importance matrix of the random forest model**.

The feature importance analysis of the RF model is shown in Fig. 3. QoL was identified as the paramount predictor of SI, followed by the number of depressive episodes, history of SAs, subjective cognitive complaints, emotional abuse from childhood trauma, TG level, age, social support, objective cognitive functioning, resilience, self-esteem, Ca^2+^ level, and RBC count.

**Fig. 4.**
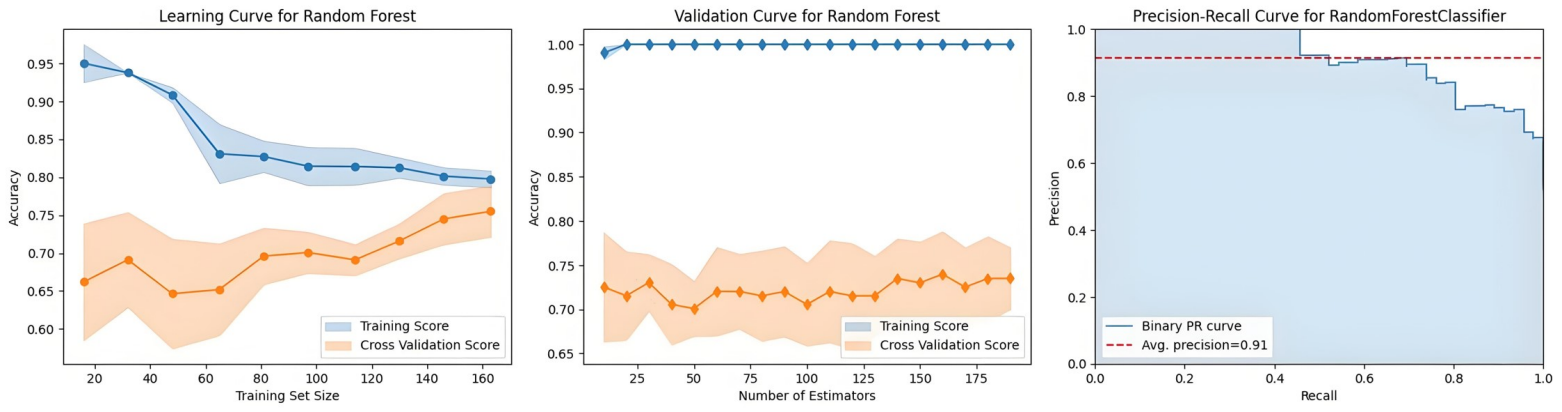
**Evaluation curves of the random forest model**.

Three evaluation curves revealed the behavioral patterns of the RF model (Fig. 4). The learning curve exhibited progressive convergence between training accuracy (modestly declining) and cross-validation accuracy (steadily increasing) with increasing dataset size, demonstrating enhanced generalization capability without overfitting. Validation profiling indicated significant performance gains with increasing estimator counts until an optimization plateau was reached at approximately n = 150, beyond which marginal improvements became statistically negligible. The precision‒recall relationship proved robust across classification thresholds, achieving an average precision score of 0.922, while maintaining clinically relevant precision (≥0.85) at recall rates exceeding 0.80, indicating reliable positive case identification capability.

Finally, an operational web-based prediction interface was developed to enable suicide risk prediction by inputting the identified risk factor profiles, thereby facilitating risk assessment in diverse health care settings.

## 4. Discussion

In this study, nine ML models were developed and compared to predict the risk of SI in patients with BD. The RF model demonstrated superior performance, achieving an AUC of 0.915 with a classification accuracy of 81.8%. Through interpretable SHAP analysis, QoL emerged as the most salient predictor, followed by the number of depressive episodes, history of SAs, cognitive functioning, and emotional abuse. These findings underscore the multifaceted nature of SI risk in BD.

Our results align with existing evidence on SI risk factors in BD [15, 44]. Machine learning approaches have also been successfully applied to identify suicide risk in other populations, such as veterans, by leveraging cross-modal interactions among psychosocial factors [45]. The multimodal ML model developed by Pigoni *et al*. [44] focused on predicting SAs within a 12-month period and found that combining clinical features with gray matter volume significantly improved predictive sensitivity (reaching 80%). This highlights the important supplementary value in predicting suicidal behavior of neurostructural features, particularly in the prefrontal-temporal-cerebellar regions. Although the present study did not include neuroimaging data, it also achieved high predictive accuracy for SI by incorporating multidimensional features such as psychosocial factors, cognitive functioning, and biomarkers, further supporting the necessity of integrating multidimensional information in suicide risk prediction. In addition, the theoretical model of suicide proposed by Malhi *et al*. [15] emphasizes that the transition from ideation to action involves multi-stage mechanisms such as motivation and volition. The importance of factors such as previous SAs and cognitive functioning in our findings corresponds to elements like acquired capability and cognitive appraisal in Malhi’s [15] model, while childhood trauma and QoL reflect the foundational roles of early stress and current psychosocial functioning in the formation of SI. Furthermore, Deng *et al*. [21] also demonstrated the utility of ML and their model achieved an AUC of 0.741 in testing and 0.788 in external validation. Notably, their SHAP analysis identified polarity (i.e., depressive vs. manic episode) and previous SAs as the most influential predictors, which resonates with the “acquired capability” component in Malhi’s model [15] and underscores the transdiagnostic relevance of these factors. Although Deng *et al*. [21] did not incorporate neuroimaging biomarkers, their model’s strong performance further validates the importance of integrating multidimensional clinical data for suicide risk prediction. In summary, by focusing on the earlier risk stage of SI, this study expands the application of ML in suicide risk prediction in BD and establishes strong theoretical and empirical connections with existing research.

Through feature importance analysis, QoL was determined to be a paramount predictor of SI. These findings are consistent with those of a study by Mazaheri *et al*. [46] involving 140 inpatients with BD type I, which demonstrated an inverse association between QoL and suicide risk. This suggests that the subjective burden of illness, encompassing functional impairment and psychosocial well-being, may be a more immediate driver of suicidal thinking than some traditional clinical metrics are. It is widely recognized that the direct disease burden of BD significantly compromises QoL through symptomatic manifestations and functional limitations. In reality, diminished QoL also serves as a clinical indicator of compromised psychosocial resources, such as inadequate social support networks and reduced cognitive reserves, all of which collectively contribute to the development of SI [47].

The number of depressive episodes was another critical predictor of SI. These findings concur with prior research demonstrating that increased depressive episodes correlate with increased suicide risk in patients with BD [48]. Recurrent depressive episodes can exacerbate hopelessness, helplessness, and perceived burdensomeness, which are well-established psychological precursors to suicide. In addition to illness characteristics, a history of SAs was also confirmed as a pivotal predictor, which is consistent with the literature [49]. For example, Jiang *et al*. [50] reported that a history of SAs was a significant predictor of suicide risk in both AdaBoost and binary logistic regression algorithms. This heightened vulnerability may arise from acquired familiarity with suicidal means and lowered aversion to death among individuals with prior SAs. With respect to developmental trajectories, younger age correlated significantly with SI, which is consistent with the literature reporting elevated suicide risk specifically among BD patients aged <35 years [51]. This association likely reflects, in part, the confluence of earlier illness onset and underdeveloped coping mechanisms coupled with limited psychosocial resources in younger individuals. Furthermore, the initial period following diagnosis represents a high-risk window for suicidal behaviors [51], offering a plausible explanation for the increased SI prevalence observed in the younger patients in this study.

Our results reinforce the evidence linking both subjective and objective cognitive deficits to SI. Neurobiologically, cognitive impairments are correlated with functional alterations in the ventral and dorsolateral prefrontal cortices, with cognitive inhibition impairment specifically recognized as a neurocognitive marker for suicide prediction [52]. With respect to the relationship between subjective and objective measures, the current evidence indicates limited convergence. Some studies propose subjective complaints as harbingers of subsequent objective decline [53], whereas others report no significant association between the two [54]. Cognitive deficits may exacerbate depressive symptoms, and depressive episodes may amplify cognitive dysfunction, collectively increasing suicide risk [14, 55]. For instance, executive dysfunction can lead to deficient inhibitory control, impulsive decision-making, emotional dysregulation, and compromised problem-solving abilities, fostering helplessness and intensifying depressive effects. Conversely, recurrent depressive episodes may further decrease cognitive capacity and the ability to suppress intrusive suicidal thoughts.

The significant contribution of emotional abuse from childhood trauma is consistent with the established literature [56]. Exposure to such trauma may foster maladaptive cognitive schemas, heightening an individual’s susceptibility to hopelessness during stressful periods and subsequently increasing their vulnerability to SI. Evidence indicates a potential dose‒response association between the extent of emotional abuse exposure and the severity of BD [57], further contextualizing its clinical relevance. Against this backdrop of vulnerability, resilience was recognized as a protective predictor against SI in our analysis, corroborating the established literature [58]. Individuals with greater resilience may preferentially adopt adaptive coping strategies (such as problem solving), thereby disrupting the progression from hopelessness to SI. An intriguing phenomenon was observed from the methodological perspective. Variables that were not statistically significant in the univariate analysis (e.g., self-esteem) exhibited relatively high |SHAP| values in the RF model. This divergence likely reflects the inherent constraints of univariate approaches, which evaluate variables linearly and independently, overlooking the potential intricate interactions among variables. In contrast, the ensemble decision trees of the RF model can capture nonlinear associations and higher-order variable interactions, potentially elucidating the nuanced relationship between self-esteem and SI.

In the present study, social support was identified as a significant predictor of SI. Existing evidence indicates divergent mechanistic pathways. Tamizi *et al*. [59] demonstrated a direct association, in which social support independently accounted for 33.3% of the variance in SI among Malaysian university students. Conversely, Owen *et al*.’s [60] four-month longitudinal study of 80 patients with BD revealed a more complex chain mediation pathway in which low social support leads to feelings of defeat, fosters entrapment cognition, and subsequently amplifies hopelessness, ultimately increasing the risk of SI.

Biochemical markers, such as TG levels, Ca^2+^ levels, and RBC counts, demonstrated the predictive potential of the SI for BD. However, mechanistic interpretations necessitate nuanced contextualization. With respect to TG levels, our analysis revealed significantly lower concentrations in SI patients, potentially reflecting disease-associated appetite suppression and decreased nutritional intake. Conversely, Liu *et al*. [61] reported that elevated TG levels (>1.7 mmol/L) were associated with an increased risk of SI (odds ratio = 2.58). This disparity can be attributed to population differences. Specifically, the latter study concentrated on children and adolescents with metabolic syndrome, which may heighten cardiovascular risks and, subsequently, vulnerability to SI. In addition to lipid metabolism, patients with SI exhibited reduced Ca^2+^ levels. This phenomenon may be attributed to lithium-induced inositol depletion and calcium channel blockade, both of which lead to a reduction in the intracellular Ca^2+^ concentration. This can potentially indicate illness chronicity and treatment duration. Concurrently, the decreased RBC counts in patients with SI align with previous findings [62], which could be ascribed to chronic low-grade inflammation in BD that suppresses erythropoiesis and shortens the lifespan of erythrocytes. Future research should explore additional biomarkers, such as neurotransmitters and inflammatory factors. For example, Li *et al*. [55] developed an optimized prediction model (AUC = 0.91) that integrated serum brain-derived neurotrophic factor (BDNF) levels, cognitive functioning, depressive symptoms, and sleep quality to predict SI in patients with bipolar depressive disorder.

### Strengths and Limitations

This study possesses several strengths. First, a comprehensive multidimensional assessment framework encompassing sociodemographic, clinical, psychological, cognitive, environmental, and functional domains was employed to allowed for a holistic exploration of predictors associated with SI in BD. Second, the adoption of a hybrid feature selection strategy integrating both data-driven (Boruta algorithm) and clinician-guided approaches enhanced the clinical relevance and interpretability of the selected predictors while maintaining statistical robustness. Third, the use of explainable ML techniques, particularly SHAP, facilitated the interpretation of complex model decisions and identified clinically actionable risk factors, thereby bridging the gap between black-box predictive models and clinical utility. Finally, the development and deployment of an interactive web-based prediction tool (http://losha:8020) demonstrate a commitment to translational research, offering a practical means for real-time risk assessment in clinical settings.

However, several limitations must be acknowledged. First, the cross-sectional design constrained causal inference and obscured the temporal dynamics of risk factors, necessitating a longitudinal study to validate predictive stability. Second, the modest sample size (n = 292), while sufficient for initial model development, may limit the stability of our findings. Future studies with larger, multicenter cohorts are essential to validate and refine this prediction model. Third, all participants were recruited from tertiary hospitals and the model lacks an external validation in independent cohorts. This may limit the model’s generalizability to other patient populations and clinical settings. Fourth, the lack of a control group (e.g., patients with major depressive disorder or healthy controls) prevents us from determining whether the identified predictors are specific to BD or represent general risk factors for suicidality across diagnostic boundaries. Fifth, the use of self-reported questionnaires may be subject to recall and social desirability biases. In addition, although a wide range of covariates were included, unmeasured confounding factors, such as detailed pharmacotherapy history and BD subtype differences, may still have affected the results. Furthermore, SI assessment involves multifaceted complexities. Although this study incorporated routine blood biochemical markers from medical records, the limited specificity and theoretical foundations have restricted their clinical interpretation. Future research should explore more targeted biomarkers, such as those associated with the gut–brain axis (e.g., neurotransmitters, immune factors, and the gut microbiota), to systematically explore the biological mechanisms underlying SI and SAs [63]. Finally, although a web-based predictive tool was developed to facilitate potential clinical translation, its performance and utility necessitate further validation in prospective, real-world clinical settings. In addition, it is crucial to note that the current version of this web tool is a research prototype. While we have implemented basic security measures (e.g., client-side data processing to avoid server transmission and no collection of personal identifiers), it has not yet undergone a formal security audit or obtained certifications for clinical use. Its full compliance with medical software regulations (e.g., from the NMPA in China or equivalent bodies elsewhere) and its resilience against sophisticated cyber threats have not been established. Therefore, any future clinical application must be preceded by a comprehensive security and compliance assessment.

## 5. Conclusions

In this study, nine ML models for predicting SI in patients with BD were developed and compared, with the RF model demonstrating the optimal performance. Interpretability analyses revealed that QoL, the number of depressive episodes, history of SAs, cognitive functioning, and childhood emotional abuse were the most influential predictors. This study, which combined hybrid feature selection with explainable ML, provides a validated framework for assessing suicide risk and highlights specific targets for clinical intervention. However, the model requires further external validation in prospective, real-world cohorts to confirm its efficacy and generalizability before it can be recommended for routine clinical use. Future research should focus on longitudinal designs to establish causal inference and on integrating these models into collaborative care settings to test their impact on patient outcomes.

## Data Availability

The data and materials used in this study are available from the corresponding author upon reasonable request.

## Abbreviations

SI, suicidal ideation; BD, bipolar disorder; SAs, suicide attempts; ML, machine learning; HDRS-17, 17-item Hamilton depression rating scale; YMRS, Young mania rating scale; BMI, body mass index; WHR, waist-hip ratio; RBC, red blood cell; WBC, white blood cell; HGB, hemoglobin; AST, glutamic oxaloacetic transaminase; ALT, glutamic-pyruvic transaminase; ALB, albumin; TP, total protein; GLO, globulin; TBIL, total bilirubin; BG, blood glucose; CHO, cholesterol; TG, triglycerides; HDL, high-density lipoprotein cholesterol; LDL, low-density lipoprotein cholesterol; APOA1, apolipoprotein A1; APOB, apolipoprotein B; COBRA, cognitive complaints in bipolar disorder rating assessment; RBD, resilience questionnaire for bipolar disorder; RSES, rosenberg self-esteem scale; CTQ, childhood trauma questionnaire; SSRS, social support rating scale; FAD, family assessment device; QoL, quality of life; SF-12, 12-item short form health survey; LR, logistic regression; XGBoost, extreme gradient boosting; LightGBM, light gradient boosting machine; CatBoost, categorical boosting; GBM, gradient boosting machine; AdaBoost, adaptive boosting; ET, extremely randomized trees; SVC, support vector classification; ROC, receiver operating characteristic; AUC-ROC, area under the receiver operating characteristic curve; MCC, Matthews correlation coefficient; PR, precision-recall; TRIPOD, transparent reporting of a multivariable prediction model for individual prognosis or diagnosis.

## Supplementary Material

Supplementary material associated with this article can be found, in the online version, at https://doi.org/10.31083/AP45589.
